# Synthesis and characterization of low density porous nickel zinc ferrites

**DOI:** 10.1039/c9ra01076a

**Published:** 2019-04-30

**Authors:** Qiushan Yu, Yuchang Su, Rabigul Tursun, Jing Zhang

**Affiliations:** School of Materials Science and Engineering, Central South University Changsha 410083 China emlink@csu.edu.cn +86 731 88830785; School of Physics and Optoelectronic Engineering, Yangtze University Jingzhou 430023 China

## Abstract

Ni–Zn ferrite has important applications in the field of soft magnetic materials due to its excellent magnetic properties, but its high bulk density hinders its promotion. Herein, an oxalate precursor was prepared by a coprecipitation method with metal sulfate and oxalic acid as raw materials. The low density porous Ni–Zn ferrite powder was prepared by thermal decomposition in an aerobic environment with the oxalate precursor. The microstructure, morphology, and dielectric and magnetic properties of Ni–Zn ferrite were studied by thermogravimetric and differential scanning calorimetry, X-ray powder diffraction, X-ray photoelectron spectroscopy, Fourier transform-infrared spectroscopy, scanning electron microscopy, transmission electron microscopy, tap density testing for powder, vibrating sample magnetometry, specific surface and aperture analysis and vector network analysis. The results showed that the purity, morphology, grain size and saturation magnetization of Ni–Zn ferrite were controlled by many factors such as synthetic temperature, retaining time and environmental conditions. Under an oxygen atmosphere, pure Ni–Zn ferrite can be prepared from an oxalate precursor by a thermal process. The ferrite has a wood-splitting appearance and a multi-layered internal cavity structure, and the bulk density is only 1/3 of the general ferrite. It has good soft magnetic and microwave absorbing properties, which makes it a potential excellent material for microwave absorbers.

## Abstract 2

Ni–Zn ferrite has important applications in the field of soft magnetic materials due to its excellent magnetic properties, but its high bulk density hinders its promotion.

## Graphical abstract

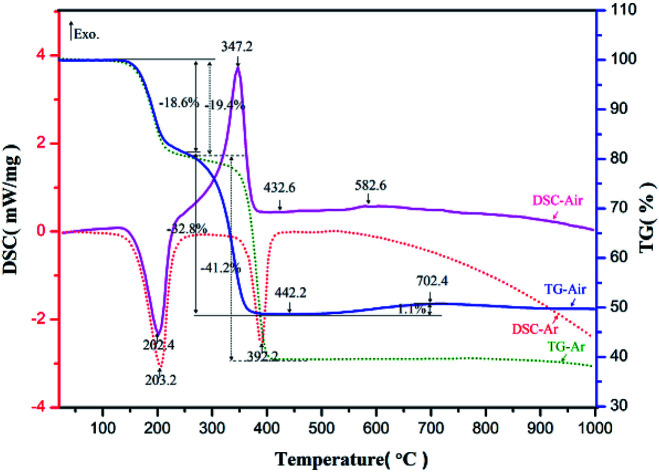

## 1. Introduction

In recent years, transition metal ferrite (MFe_2_O_4_) nanomaterials have been the focus of scientific research due to their superior physical properties and potential applications in magnetic devices.^1–4^ Among them, Ni–Zn ferrite (Ni_*x*_Zn_1−*x*_Fe_2_O_4_, NZFO) has become one of the most important soft magnetic materials due to its high saturation magnetization and resistivity and low coercivity.^5–9^ NZFO has become a leader in the research field of soft magnetic materials, and it is widely used in electronic, magnetic and optical fields, such as high frequency inductance magnetic cores, transformers, magnetic recording materials, and microwave absorption. However, the shortage of high density of traditional Ni–Zn ferrite significantly restricts its popularization and application in some fields.^10–12^

Ni–Zn ferrites can be synthesized using a variety of methods, including laser deposition,^13^ coprecipitation,^14^ ball milling,^15^ sol–gel,^16^ hydrothermal,^17^ self-propagating combustion,^18^ precursor,^19^ ultrasonic cavitation,^20,21^ and microemulsion^22^ methods. In order to solve the problem of over high density, the present research of Ni–Zn ferrites mainly focuses on the combination and hollowing process. For example, the Ni–Zn ferrite and bamboo-charcoal are combined to effectively reduce the bulk density and achieve a good performance in wave absorption.^23^ The nanoscale ferrite powder with a bulk density of only 4.03–4.32 g cm^−3^ can be produced using hydrothermal template method with sulphate of nickel, zinc and iron as raw materials, triethylamine and polyethylene glycol as template.^24^ The diatomite/Ni–Zn ferrite composite was prepared by the sol–gel method, the density was reduced to 3.2–3.8 g cm^−3^, and the dielectric and magnetic losses were within the 1–1000 MHz frequency band.^25^ However, in view of the existing preparation methods, the hollow template is still easy to break, the process is complex, and the cost of preparation and the density of the products is still high, so they need to be further improved.

In order to solve the shortcomings of the existing technologies, a method of preparing oxalic acid compound salt precursor by coprecipitation and roasting precursors in air to obtain Ni–Zn ferrite is provided. This method has the advantages of lower density, simpler process, lower preparation cost and better wave absorbing property.

## 2. Experimental details

### 2.1 Preparation of materials

FeSO_4_·7H_2_O, ZnSO_4_·7H_2_O, NiSO_4_·6H_2_O and H_2_C_2_O_4_·2H_2_O were used as synthetic raw materials. The pH value was adjusted using dilute NH_3_·H_2_O, while hexadecyl trimethyl ammonium bromide (CTAB) was used as a dispersant. The NZFO precursor was synthesized by coprecipitation method and NZFO powder was prepared by thermal decomposition in oxygen atmosphere. All the reagents used are commercially available and were analytical grade. Deionized water was used without further purification.

In a typical preparation, 0.055 mol NiSO_4_·6H_2_O, 0.045 mol ZnSO_4_·7H_2_O, and 0.20 mol FeSO_4_·7H_2_O were dissolved in 200 mL deionized water and stirred until completely dissolved. Next, 0.5 g CTAB and 0.32 mol H_2_C_2_O_4_·2H_2_O were dissolved in 500 mL deionized water, and a diluted NH_3_·H_2_O was added with magnetic stirring to adjust the pH value. Under continuous magnetic stirring at 600 rpm, the oxalic acid solution was heated to a preset temperature of 80 °C and kept at a constant temperature, then the mixed sulfate solution was pumped into the solution at a rate of 15 mL min^−1^ using a peristaltic pump, which resulted in yellowish turbidity. The solution was held at this temperature for 2 h, then cooled to room temperature naturally and filtered. The filter residue was cleaned with deionized water and anhydrous ethanol rinse three times, dried for 24 h at 60 °C to obtain the oxalate precursor. The precursor was then heated to 450 °C at a rate of 2 °C min^−1^, and kept at 450 °C for 30 min, and then heated to 500–800 °C at a rate of 5 °C min^−1^ and kept for 2 h for heat preservation, and then naturally cooled with the furnace to obtain the final nickel zinc ferrite powder.

### 2.2 Characterization and testing of materials

To test and characterize the properties and structures of the samples, thermogravimetric and differential scanning calorimetry (TG-DSC) analyses were carried out using a simultaneous thermal analyzer (STA449C, NETZSCH) from 25 °C to 1000 °C at a heating rate of 10 °C min^−1^. The crystalline phase of the powders was characterized using X-ray diffraction (XRD) at room temperature with a Rigaku D/Max 2500 powder diffractometer with Cu K_α_ radiation (*λ* = 1.5406 Å) at a scanning rate of 8° min^−1^ in the 2*θ* range of 10–80°. Sample structure bonding was characterized by Fourier-transform infrared (FTIR) spectroscopy (Nicolet Nexus) over a range of 400–2000 cm^−1^ and the magnetic properties of the pure NZFO samples were characterized using a vibrating sample magnetometer (VSM, Lake Shore 7410) within 1 T magnet at room temperature. Characterization and determination of the valence state of iron elements obtained by XPS(Escalab 250Xi). Scanning electron microscopy (SEM, FEI SIRION 200 and Nano SEM 450) and Transmission electron microscope (TEM, FEI Tecnai G2 F20) was used to assess the micromorphology of the NZFO powders and vibration density tester (VDT, WuLing TW-01) was used to obtain the density of powders. Then, surface area and porosity analyzer (Micromeritics, ASAP2460) was used to obtain the adsorption–desorption curve and mesoporous distribution curve. The dielectric and magnetic losses of the sample were analyzed by AV3672B–S vector network analyzer.

## 3. Results and discussion

### 3.1 Thermal analysis

**Fig. 1:**
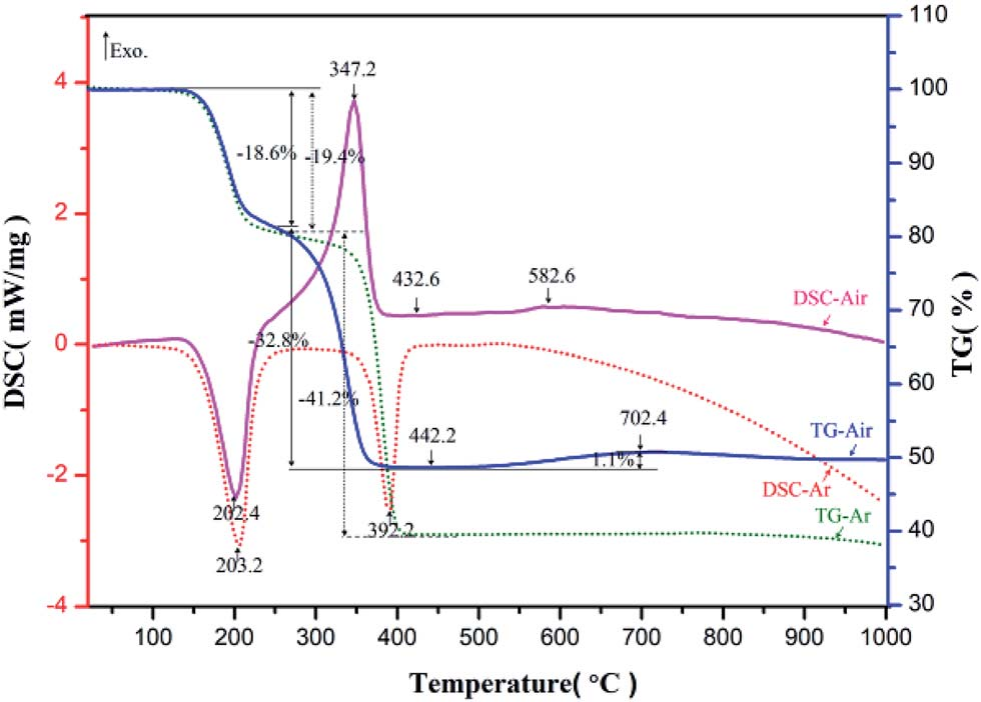
TG-DSC curves of Ni–Zn ferrioxalate precursor.

The prepared nickel–zinc–iron oxalate precursors were analyzed by TG-DSC in argon and air respectively. The obtained curves are shown in Fig. 1.

In argon, the first endothermic peak appeared near 203.2 °C and about 19.4% of the mass was lost. Based on the analysis of the existing literature and infrared spectrum,^26^ it is believed the crystalline water was lost after the molecules were heated (Formula (1), M represents a transition metal element), where the loss rate of 19.4% is very close to the theoretical value of 19.7%. The second endothermic peak appeared near 392.2 °C and the mass decreased by about 41.2%, which is conjectured to be the theoretical mass loss of more than 39.6% during decomposition of Ni–Zn ferrioxalate precursor into metal oxide, carbon monoxide, carbon dioxide (Formula (2)) and reduction of some metal oxides into metal monoxide or intermetallic compounds (Formula (3)). In air, the first heat absorption peak appeared near 202.4 °C and the loss of crystal water was about 18.6% (Formula (4)). The exothermic peak was near 347.2 °C, which is when CO and CO_2_ gases were released from the oxalate and CO was quickly oxidized by O_2_ in the surrounding air and heat was released (Formula (5)). The mass reduction was about 32.8%, which is less than the 39.6% weight loss in the argon environment, suggesting that the precursor was not reduced to a single or intermetallic compound with metal oxides in an aerobic environment (Formula (5)). At 520–720 °C, the mass of the sample increased slowly by 1.1% and there was a slight exothermic peak near 582.6 °C, which may be related to oxidation of the Fe^2+^ to Fe^3+^ in the compound (Formula (6)).

1 MC_2_O_4_·2H_2_O → MC_2_O_4_ + 2H_2_O↑ − Q_T1_ (Ar, 170–220 °C)

2 MC_2_O_4_ → MO + CO↑ + CO_2_↑ − Q_T2_ (Ar, 345–425 °C)

3 MO + CO → M + CO_2_↑ − Q_T3_ (Ar, 345–425 °C)

4 MC_2_O_4_·2H_2_O → MC_2_O_4_ + 2H_2_O↑ − Q_T4_ (Air, 170–220°C)

5 2MC_2_O_4_ + O_2_ → 2MO + 4CO_2_↑ + Q_T5_ (Air, 345–425°C)

6 O_2_ + MFeO → MFe_2_O_4_ + Q_T6_ (Air, 520–720°C)

According to changes in TG-DSC curve characteristics, it can be deduced that pyrolysis of the oxalate sample can be divided into three steps. First, the crystalline water was lost near 203 °C. Then, the oxalate decomposed into tiny grains of iron oxide, zinc oxide, and nickel oxide at 345–425 °C, where they mixed uniformly and were in contact with each other and the interfacial resistance between the crystals was low. Finally, in the range of 520–720 °C, each tiny grain of solid state reaction was capable of generating NZFO at a low temperature. Analysis of samples using XRD and FT-IR spectroscopy supports these inferences.

### 3.2 Structural analysis

**Fig. 2:**
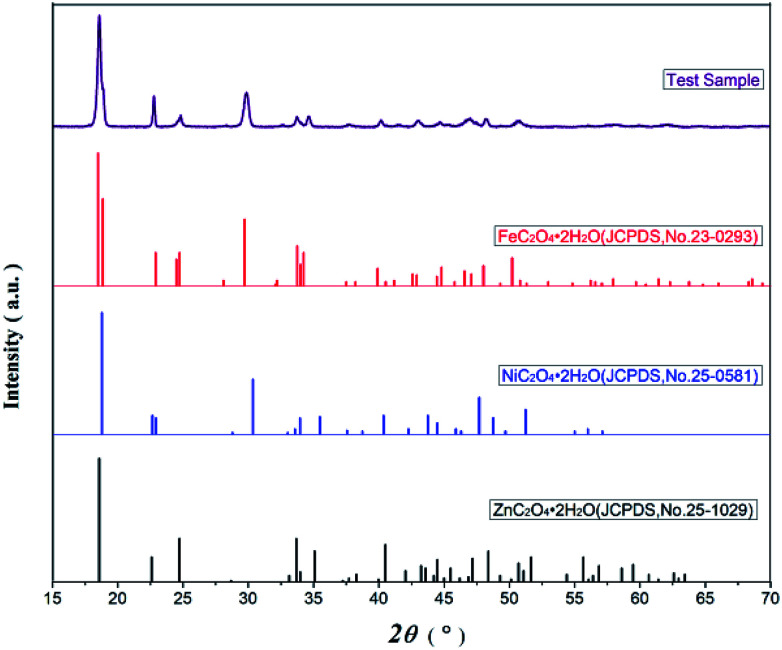
The XRD patterns of Ni–Zn ferrioxalate precursors and corresponding oxalate.

Fig. 2 presents the XRD spectra of oxalate precursor, FeC_2_O_4_·2H_2_O (JCPDS no. 23-0293), ZnC_2_O_4_·2H_2_O (JCPDS no. 25-1029), and NiC_2_O_4_·2H_2_O (JCPDS no. 25-0581). The latter three contain the same number of spatial groups (no. 15) in their crystal structures and belong to the monoclinic system. The Fe has an atomic radius similar to Ni and Zn, while the characteristic XRD peak of the oxalate precursor was similar to the latter three compounds. However, there were no single FeC_2_O_4_·2H_2_O, ZnC_2_O_4_·2H_2_O, or NiC_2_O_4_·2H_2_O peaks, combined with infrared spectroscopy, indicating the Fe^2+^, Zn^2+^ and Ni^2+^ in the oxalate precursor have been coprecipitated. The precursor was a complex, rather simple, mixture of ferrous oxalate and oxalic acid nickel or zinc oxalate.

**Fig. 3:**
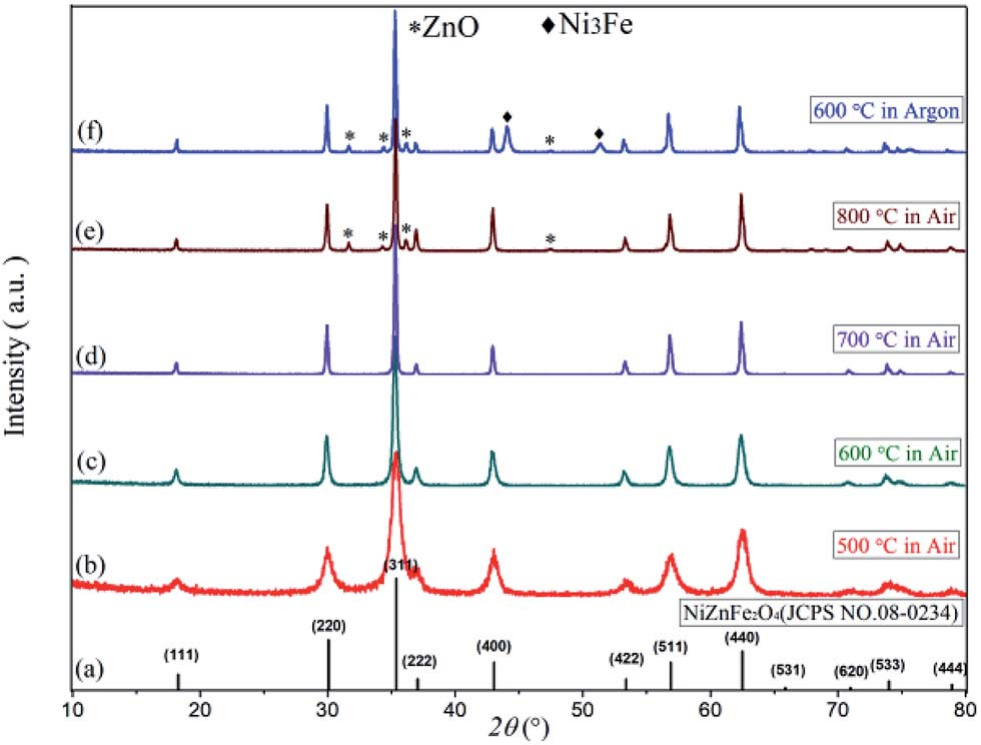
The XRD patterns of (a) Ni_0.5_Zn_0.5_Fe_2_O_4_ standard diffraction pattern, (b)–(f) Ni_0.55_Zn_0.45_Fe_2_O_4_ diffraction pattern.

Fig. 3 displays the XRD diffraction patterns and standard spectra of precursor samples after calcination for 2 h at 500–800 °C. The obtained XRD diffraction patterns for each sample contain the diffraction peak of Ni_0.5_Zn_0.5_Fe_2_O_4_ (JCPDS file no. 08-0234) with the spinel structure: at the 2*θ* diffraction angles 18.3°, 30.1°, 35.4°, 37.1°, 43.0°, 53.4°, 56.9° and 62.5°, there were peaks reflective of crystalline surfaces with (111), (220), (311), (222), (400), (422), (511) and (440). There were no obvious heterozygous peaks in the diffraction patterns after 2 h at 500–700 °C in the air, indicating that the samples had good purity. Conversely, there were still some clutter peaks after 2 h at 600 °C in argon gas. Based on these data, combined with the TG-DSC curve analysis from Fig. 1, Ni–Zn ferrites can be synthesized at 500 °C. Because ZnO or other heterozygous phases can be found at 800 °C, the pure phase can be synthesized only at 600–700 °C and an environment containing oxygen is necessary for obtaining pure NZFO.

The average sizes of the crystal grains in the samples can be calculated using Debye–Sherr's formula:^27^7
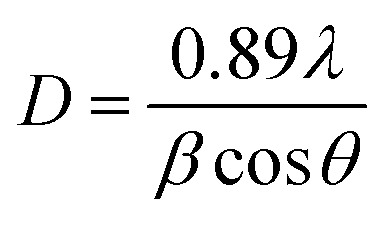
where *D* is the crystal grain size, 0.89 is Sherr's constant, *λ* = 1.5406 Å is the wavelength of the X-ray Cu K_α_source, *β* is the width at half-maximum of the diffraction peaks, and *θ* is the diffraction angle. The average crystal grain size in the samples ranged from 10.6 ± 0.4 to 58.6 ± 0.9 nm. During treatment, the crystal grew gradually as the temperature increased. In order to obtain a smaller crystal grain size, treatment has to be performed at a lower temperature.

### 3.3 FT-IR analysis

**Fig. 4:**
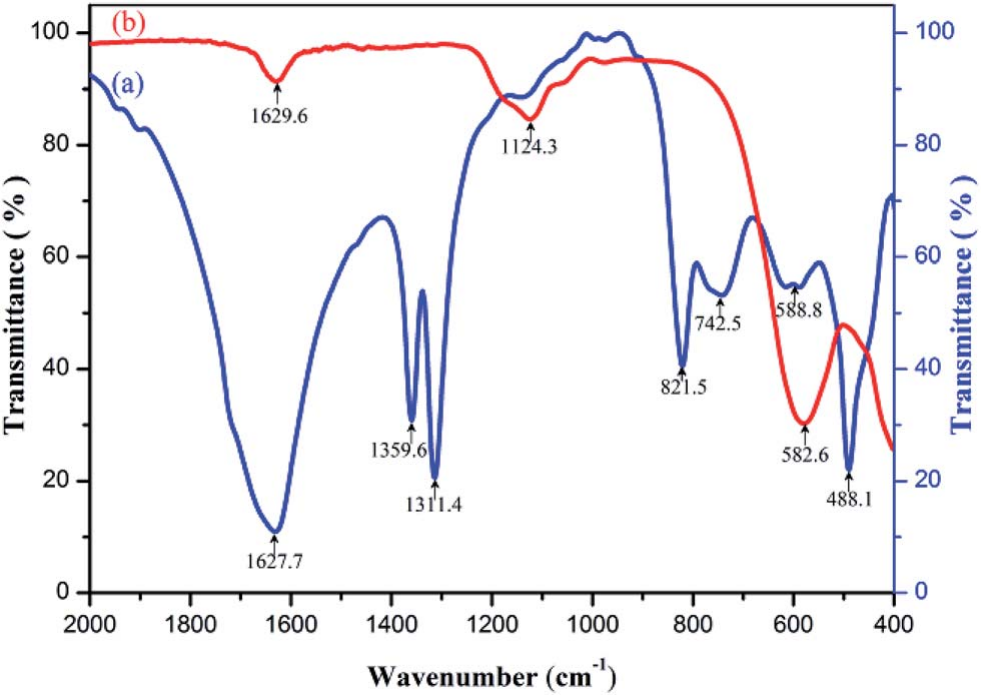
Infrared spectra of (a) Ni–Zn ferrioxalate precursor, (b) Ni_0.55_Zn_0.45_Fe_2_O_4_ obtained by calcining the precursor at 600 °C for 2 h.

The samples of precursor and calcinator were mixed with KBr and pressed into tablets respectively. The infrared spectra with wavenumber of 400–2000 cm^−1^ were measured by FT-IR spectrometer. The curves (a) and (b) were obtained as shown in Fig. 4. The strong absorption peaks near 1360 cm^−1^ and 1312 cm^−1^ indicate that a large number of OH radicals existed in the curves (a),^28^ but disappeared in the curves (b), indicating that the OH radicals in the precursors were removed after calcination. In addition, the oxalate precursor lost the crystalline water; the same phenomenon also appeared in the curve (a) around 822 cm^−1^ C–C bending vibration absorption peak,^28^ after heat treatment disappeared in the curve (b), indicating that oxalate was removed in heat treatment, which can be verified by TG-DSC and XRD test results. There were two strong characteristic absorption peaks in the 550–670 cm^−1^ of the curve (a), which should be caused by the stretching vibration of Zn–O, Ni–O and the winding vibration of Fe–O in the tetrahedron.^29–33^ After burning, the two peaks disappeared completely, and a new single absorption peak of metal oxides appeared near 583 cm^−1^ in the curve (b). XRD and Energy Dispersive Spectrometer (EDS) analysis showed that the two peaks were from Ni–Zn ferrite. In addition, there were some stretching vibration absorption peaks near 1124 cm^−1^ and some stretching vibration absorption peaks near 488 cm^−1^, which may be related to the residual sulfate in the sample.

### 3.4 XPS analysis

In Ni–Zn ferrite, the valence state of iron element can affect its ability to occupy the B site in the octahedron or the A site in the tetrahedron, which will affect the crystal structure, properties and bulk density of the ferrite. We used XPS to characterize and analyze the valence state of iron element in the compound. The XPS peaks obtained from the samples were calibrated by the C1s peak at 284.68 eV, and the Lorentz function was used to fit and analyze the peaks of the elements in the spectrum.

**Fig. 5:**
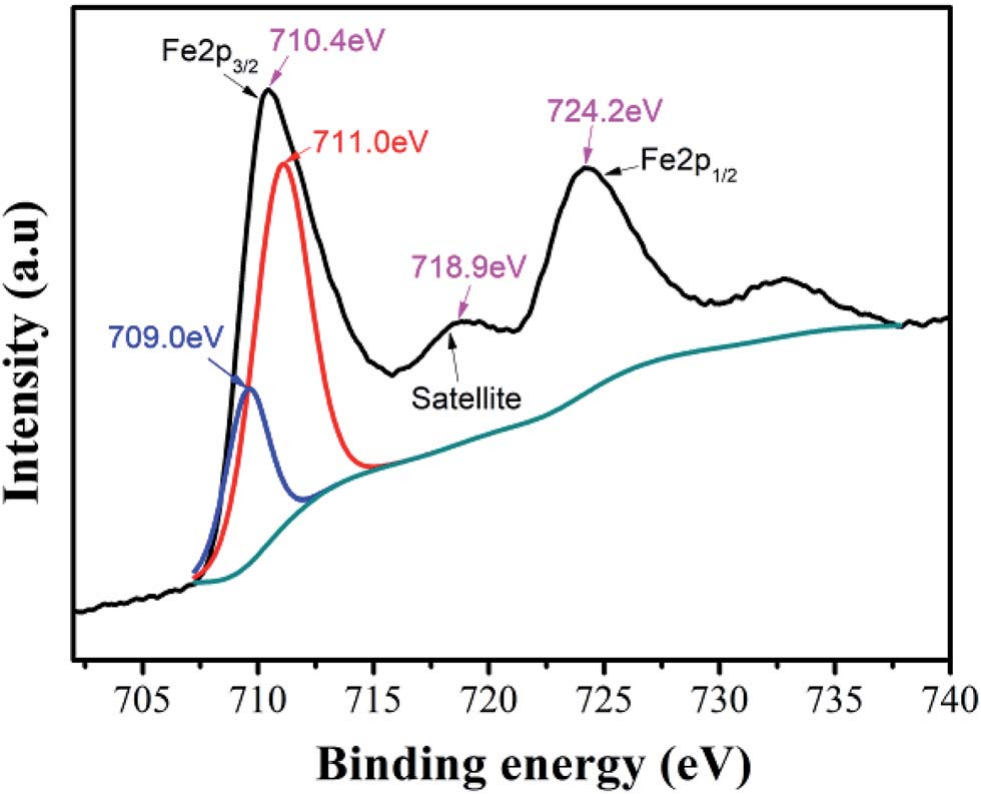
Iron ion XPS spectra of Ni–Zn ferrite.

In the XPS band spectrum of the sample, the binding energy interval of 705.0 eV to 730.0 eV is one of the intervals in which the characteristic spectrum of iron element is located,^34^ as shown in Fig. 5. There are spin-orbital energy peaks belonging to Fe 2P_3/2_ and Fe 2P_1/2_ near the two positions of 710.4 eV and 724.2 eV. These values of spin-orbital binding energy are slightly lower than Fe 2P_1/2_ (724.4 eV) and Fe 2P_3/2_ (711.0 eV) of Fe_2_O_3_, but higher than Fe 2P_1/2_ (722.6 eV) and Fe 2P_3/2_ (709.0 eV) of Fe_2_SiO_4_.^35^ This implies that they may be composite peaks formed by Fe^2+^ and Fe^3+^ ions in the vicinity of the Fe 2P_3/2_ orbital. To this end, the Fe 2P_3/2_ peak near 710.4 eV was fitted to the peak, while the two peaks with peak values of 711.0 eV and 709.0 eV were successfully separated, indicating that the speculation is correct. The compound contains both Fe^2+^ and Fe^3+^ ion.

### 3.5 SEM and TEM characterization

**Fig. 6:**
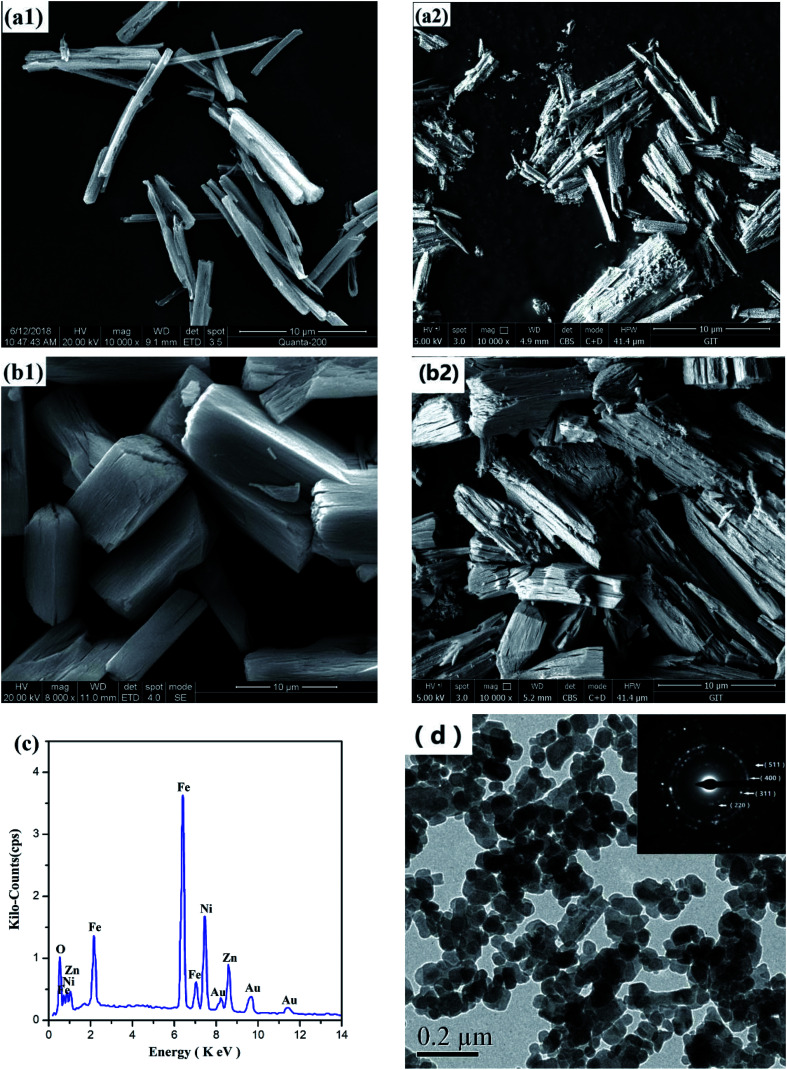
SEM images of Ni–Zn oxalate precursor under (a1) 80 °C, (b1) 60 °C and corresponding Ni–Zn ferrites (a2), (b2); (c) EDS diagram of NZFO in (a2); (d) TEM images and SAED of Ni–Zn ferrite.

Fig. 6(a) and (b) shows the SEM morphology of two Ni–Zn oxalate precursors and Ni–Zn ferrites. They are all wood-splitting shapes, and the ratio of radial to axial increases with the decrease of temperature.

**Fig. 7:**
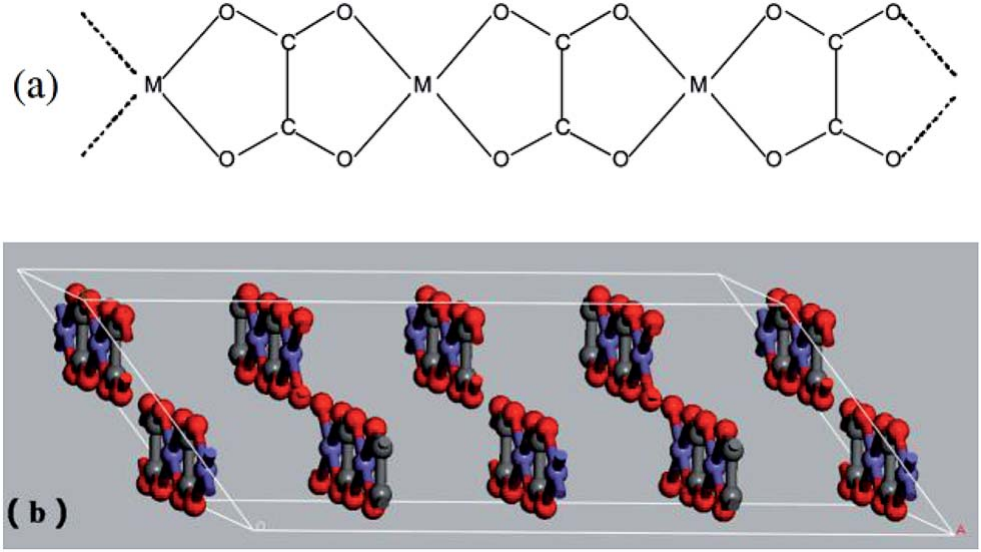
(a) Schematic drawing of infinite chain arrangement present in Fe^2+^ oxalates of MFeC_2_O_4_·*n*H_2_O; (b) the layered structure of MFeC_2_O_4_·*n*H_2_O.

The prepared Ni–Zn–Fe oxalate compounds was a monoclinic system, where its metal cations (M^2+^ = Fe^2+^, Ni^2+^, and Zn^2+^) and oxalic acid radical groups formed an infinite chain, as shown in Fig. 7(a). The oxalate radical group played the role of the four-tooth ligand here. M^2+^ was connected to two H_2_O, forming one deformed MO_6_ octahedra environment parallel to the *b* axis and formed a regular arrangement in the layered structure. The layered structure was perpendicular to the *c* axis, as shown in Fig. 7(b). Therefore, the oxalate compound had an anisotropic structure along the *b* axis and the one-dimensional characteristics of the chain structure greatly influenced anisotropic growth of them.^36^ The oxalate complex had a similar chain structure, but these chain structures became mixed when formation is laminar, which rearranges hydrogen bonds between layers.^2^ When the pH value is low, there is a higher concentration of H^+^ in the solution and the oxalic acid compound salt chain structure has a relatively greater role. Therefore, the growth rate was faster in this direction, leading to formation of a rod shape, and the increase of temperature helps to strengthen this advantage (Fig. 6(a)). The rapid extension and growth of the oxalate molecular chain hinders the close bonding between the layers to some extent, so that the precursors generate more voids after the loss of organic matter during the roasting process, and become relatively more delicate.

Fig. 6(d) is a TEM photograph and a SAED (selected area electron diffraction) pattern of the sample in Fig. 6(a2). It can be seen from the figure that the sample particles are mainly spheroidal nano-grains, and there are many voids between the grains, and some grains are agglomerated. The grain size is slightly larger than the calculated value of XRD (see Table 2). The crystal faces corresponding to the electron diffraction patterns of the crystal grains are (220), (311), (400), (511), *etc.*, which are consistent with the XRD analysis results described above.

In order to obtain a point on the surface of sample in Fig. 6(a2) and quantitatively determine the elements by EDS, the target element content (Atomic%) of the sample determined by ZAF modification method is Fe : Ni: Zn ≈ 56.2 : 14.4 : 11.5 ≈ 2 : 0.51 : 0.41, which is close to the preset metal element ratio of Ni_0.55_Zn _0.45_Fe_2_O_4_ (Fig. 6(c)).^37^

### 3.6 Density measurement and pore size distribution characterization

The porous structure of nickel–zinc ferrite helps to reduce its bulk density. There are many holes on the surface of ferrite, and there are residual holes in the cross section (Fig. 6(a2) and (b2)). Because of the porous structure of Ni–Zn ferrite, and the density of the powder measured by the tap density method is only 0.76–0.78 g cm^−3^. It is about 1/3 to general Ni–Zn ferrite at 2.3 g cm^−3^ (Table 1).^24,25^

**Table 1:** Density determination of Ni–Zn ferrite prepared under different conditions

		Sample 1			Sample 2		
Preparatory conditions for precursors		pH = 3, 80 °C, 2 h			pH = 3, 60 °C, 2 h		
Project		Mass (g)	Volume (cm^3^)	Density (g cm^−3^)	Mass (g)	Volume (cm^3^)	Density (g cm^−3^)
Measurement times	1	2.571	3.43	0.750	2.530	3.21	0.788
	2	2.424	3.22	0.753	2.163	2.85	0.759
	3	2.918	3.81	0.766	2.688	3.42	0.786
Average value				0.756			0.778

**Table 2:** Grain size and magnetic parameters (*M*_s_, *M*_r_, *H*_c_ and *M*_r_/*M*_s_) of NZFO samples at room temperatures

Sample number	Heat preservation time	Grain size (nm)	*M*_s_ (emu g^−1^)	*M*_r_ (emu g^−1^)	*H*_c_ (Gauss)	*M*_r_/*M*_s_
NZFO-500	2 h	10.6 ± 0.4	9.564 ± 0.06	1.125 ± 0.01	83.102 ± 0.04	0.118
NZFO-600	2 h	20.5 ± 0.4	33.193 ± 0.09	1.638 ± 0.02	35.676 ± 0.05	0.049
			36 (ref. 40)		167 (ref. 40)	
NZFO-600-1	3 h	34.7 ± 0.5	57.946 ± 0.07	9.033 ± 0.01	83.224 ± 0.03	0.156
NZFO-700	2 h	29.2 ± 0.7	43.761 ± 0.06	4.128 ± 0.02	77.426 ± 0.03	0.094
NZFO-800	2 h	58.6 ± 0.9	55.934 ± 0.09	5.480 ± 0.01	84.024 ± 0.05	0.098

**Fig. 8:**
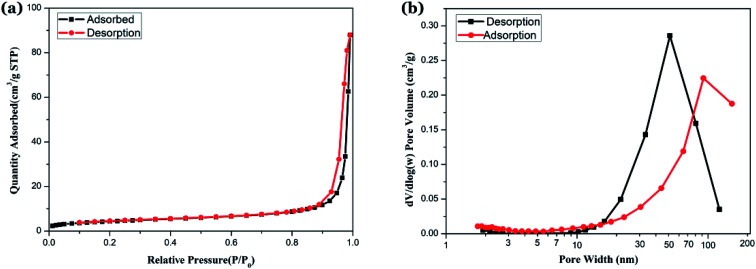
Isothermal adsorption characteristics and pore size distribution characterization of samples (a) N_2_ adsorption–desorption isotherm curves of sample; (b) BJH pore size distribution curves of sample.

In order to identify the nature of the pores and low bulk density formed in NZFO, the samples were vacuum treated at 150 °C for 10 h to remove moisture, and then the N_2_ adsorption–desorption isotherms of the samples were measured by a multi-purpose adsorption instrument. The specific surface area and pore volume were calculated by Barrett–Emmett–Teller (BET) and Barrett–Joyner–Halenda (BJH) method, and the results are shown in Fig. 8(a) and (b).

The N_2_ adsorption–desorption isotherm curves are shown in Fig. 8(a). In the low pressure section, the adsorption amount increases gently, and the adsorption amount increases rapidly when *P*/*P*_0_ is between 0.3 to 0.9, and the adsorption curve becomes steep when *P*/*P*_0_ is between 0.9 to 1.0. The curve change indicate that the sample has characteristics of a class IV curve.^38^ The desorption branch is located above the adsorption branch, and both tend to be parallel in the vicinity of the saturated vapor pressure when *P*/*P*_0_ is between 0.9 to 1.0, forming a hysteresis loop with H3 characteristics.^39^ By characterizing the morphology of the sample and the BJH aperture algorithm using TEM and SEM, we believe that the grains in the NZFO powder are loose and accumulate into mesopores with multi-layered sheet and a pore size of 50 to 80 nm (Fig. 8(b)). BET surface area is 15.85 m^2^ g^−1^, and BJH adsorption cumulative surface area of pores between 1.70 nm to 300.00 nm width is 13.30 m^2^ g^−1^, while BJH Desorption cumulative surface area of pores is 13.01 m^2^ g^−1^. These features are formed due to the following reasons: (1) the oxalate precursor is slowly oxidized during the slow heating process, and the gas is released, which leads to the formation of a large number of pore channels; (2) the organic matter in the precursor is oxidized and disappears, volume shrinks, and numerous voids between the grains form, which makes the grains loose and contributes to the formation of pores. These pores are beneficial to the improvement of microwave absorption performance and the decrease of ferrite body density.

### 3.7 Magnetic analysis

**Fig. 9:**
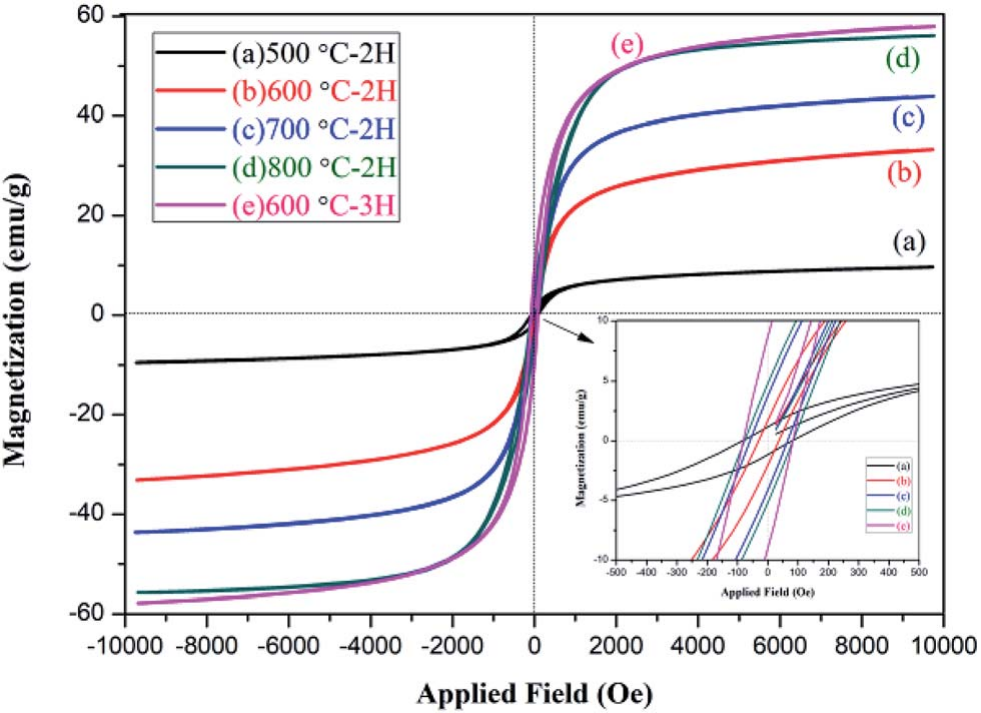
*M*–*H* loops of Ni_0.55_Zn_0.45_Fe_2_O_4_ nanocrystalline ferrites.

The magnetic properties of the NZFO nanopowders were studied by VSM magnetometer. The observed hysteresis loop (*M*–*H*) curve is shown in Fig. 9. The relative magnetic properties, such as saturation magnetization (*M*_s_), coercive force (*H*_c_), and remanent magnetization (*M*_r_) are shown in Table 2.

As shown in Fig. 9 and Table 2, the NZFO samples prepared by coprecipitation displayed obvious hysteresis behavior and the *M*_s_, *M*_r_, and *H*_c_ of NZFO-600 were 33.2 emu g^−1^, 1.64 emu g^−1^, and 35.6 gauss, respectively. The soft magnetic properties were close to or superior to those prepared using the sol–gel method by J. P. Gao (600 °C NZFO, *M*_s_ 36 emu g^−1^, and *H*_c_ 167 gauss).^40^ As the temperature increased, the *M*_s_, *M*_r_, and *H*_c_ of NZFO gradually increased, but the *M*_r_/*M*_s_ first decreased and then increased. This latter phenotype can be attributed to improvements in sample crystallinity, increases in grain size, and decreases in the number of spin suspension bonds on the surface, which lead to increases in the net spin moment and *M*_s_. When the size was larger than the critical grain size, the domain wall formed gradually and the coercive force decreased as the domain wall formed.^41,42^ With the prolongation of heat preservation time, the grain size increased along with the *M*_s_, *M*_r_, *H*_c_ and *M*_r_/*M*_s_. An increase in the latter three parameters is not conducive to the application of soft magnetic materials. NZFO-600 has the minimum remanence ratio and a narrow hysteresis loop, showing soft magnetism, a high remanence ratio, and a small coercive force can improve magnetic and electrical coupling, making it more advantageous in application in high frequency soft magnetic mediums.^43^

### 3.8 Analysis of microwave absorbing properties

**Fig. 10:**
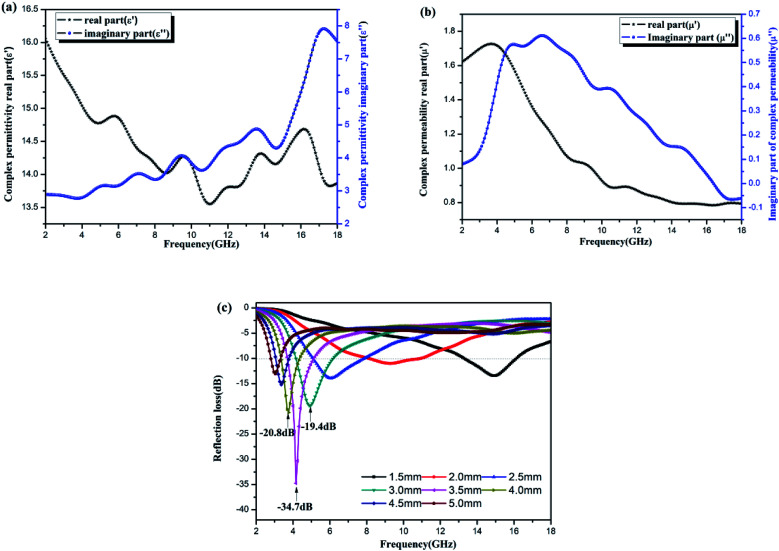
Electromagnetic parameters and wave absorbing properties of NZFO. (a) The real part (*ε*′) and imaginary part (*ε*′′); (b) the real part (*μ*′) and imaginary part (*μ*′′), (c) the reflection loss curve with frequency.

The electromagnetic properties of the Ni–Zn ferrite samples were tested by a vector network analyzer. The results are shown in Fig. 10(a)–(c). The dielectric properties are expressed by complex permittivity, the real part is the storage capacity of electric energy, and the imaginary part is the consumption capacity of electric energy.^44,45^ Magnetic properties can be expressed as complex permeability, real part as magnetic energy storage capacity, and imaginary part as magnetic energy consumption capacity.^2,44^

Fig. 10(a) is a diagram of the dielectric constant of Ni–Zn ferrites with frequency. The real part and imaginary part are about two times the real and imaginary parts of the diatomite/Ni–Zn ferrites prepared by sol–gel method,^25^ indicating that the samples have stronger dielectric parameters than the latter. Fig. 10(b) shows the frequency dependent permeability curve of Ni–Zn ferrite sample. The real and imaginary values of permeability are weaker than those of diatomite/Ni–Zn ferrite (literature). Fig. 10(c) shows the reflection spectrum of the sample to 2–18 GHz microwave. When the film thickness is 5.0 mm, the sample has an extreme value of −34.7 dB nearby the 4.2 GHz, and the reflectivity of other thicknesses of films is mostly lower than −5 dB. The microwave absorption performance of the films is better than that of diatomite/Ni–Zn ferrite.^25^

The electromagnetic properties of spinel ferrite mainly depend on the distribution of metal ions in the crystal structure and the spatial structure of the grain. In spinel ferrites, oxygen ions locate in cubic dense packing, and metal ions are distributed in the oxygen ion gap, as shown in the following Formula (8):

8 (Me_*δ*_^2+^Fe_1−*δ*_^3+^)[Me_1−*δ*_^2+^Fe_1+*δ*_^3+^]O_4_

The ions in ( ) occupy position A (tetrahedral gap), and the ions in [ ] occupy position B (octahedral gap). In Ni_0.55_Zn_0.45_Fe_2_O_4_, the order of the ability of metal ions to occupy position A is Zn^2+^ > Fe^3+^ > Ni^2+^, so the cation distribution is (Zn_0.45_^2+^Fe_0.5_^3+^)[Ni_0.55_^2+^Fe_1.5_^3+^]O_4_, forming spinel ferrite.^46^ With the addition of Zn^2+^, the tendency of metal ions occupying the A site increases, some Fe^3+^ occupying the A site will enter the B site. As Zn^2+^ is a non-magnetic ion, the magnetic moment of the A site ion decreases, while the magnetic moment of the B site ion increases, which makes the magnetic moment of Ni_0.55_Zn_0.45_Fe_2_O_4_ increase compared with that of the undoped Zn^2+^. The increase of magnetic moment can increase the saturation magnetization and further enhance the permeability of the material. However, the large number of holes left by the removal of organic matter during the burning process on one hand enhances the specific surface area of microwave absorption and increases the dielectric absorption capacity, but on the other hand, the hollow structure is not conducive to the formation of large magnetic domains, hindering the transmission of magnetic and making the permeability of the sample slightly less than diatomite/Ni–Zn ferrite.

## 4. Conclusion

Ni–Zn ferrioxalate precursors with different particle size ratios were prepared by coprecipitation method with controlled temperatures and time. High-purity Ni_0.55_Zn_0.45_Fe_2_O_4_ powders with porous cavity structure were prepared by calcining the precursors in oxygen atmosphere at 600–700 °C for 2 h. The density of NZFO powders with porous chopper-like structure is only 0.76–0.78 g cm^−3^, which is about 1/3 of the density of general Ni–Zn ferrite. The microwave absorbing property test shows that the samples have good dielectric properties and low reflectivity up to −34.7 dB. It is a kind of microwave absorbing agent with low density and good absorbing property, and the test results of TEM, XPS and N_2_ adsorption–desorption isotherms show that the excellent performance is derived from the presence of a large number of mesopores in the ferrite. In addition, other transition ferrites, including porous nanoscale CoFe_2_O_4_, NiFe_2_O_4_, and other three-element and multiple metal oxide materials, can also be prepared using this method.

## Conflicts of interest

There are no conflicts to declare.

## Supplementary Material
